# DNA Motifs and an Accessory CRISPR Factor Determine Cas1 Binding and Integration Activity in *Sulfolobus islandicus*

**DOI:** 10.3390/ijms231710178

**Published:** 2022-09-05

**Authors:** Tao Liu, Ying Xu, Xiaojie Wang, Qing Ye, Zhenzhen Liu, Zhufeng Zhang, Jilin Liu, Yudong Yang, Xu Peng, Nan Peng

**Affiliations:** 1 State Key Laboratory of Agricultural Microbiology, Hubei Hongshan Laboratory, College of Life Science and Technology, Huazhong Agricultural University, Wuhan 430070, China; 2 Antibiotics Research and Re-Evaluation Key Laboratory of Sichuan Province, Sichuan Industrial Institute of Antibiotics, School of Pharmacy, Chengdu University, Chengdu 610106, China; 3 Danish Archaea Centre, Department of Biology, University of Copenhagen, Ole Maaløes Vej 5, DK-2200 Copenhagen, Denmark

**Keywords:** CRISPR adaptation, leader, Cas1 integrase, DNA motifs, accessory factor

## Abstract

CRISPR-Cas systems empower prokaryotes with adaptive immunity against invasive mobile genetic elements. At the first step of CRISPR immunity adaptation, short DNA fragments from the invaders are integrated into CRISPR arrays at the leader-proximal end. To date, the mechanism of recognition of the leader-proximal end remains largely unknown. Here, in the *Sulfolobus islandicus* subtype I-A system, we show that mutations destroying the proximal region reduce CRISPR adaptation in vivo. We identify that a stem-loop structure is present on the leader-proximal end, and we demonstrate that Cas1 preferentially binds the stem-loop structure in vitro. Moreover, we demonstrate that the integrase activity of Cas1 is modulated by interacting with a CRISPR-associated factor Csa3a. When translocated to the CRISPR array, the Csa3a-Cas1 complex is separated by Csa3a binding to the leader-distal motif and Cas1 binding to the leader-proximal end. Mutation at the leader-distal motif reduces CRISPR adaptation efficiency, further confirming the in vivo function of leader-distal motif. Together, our results suggest a general model for binding of Cas1 protein to a leader motif and modulation of integrase activity by an accessory factor.

## 1. Introduction

Clustered Regularly Interspaced Short Palindromic Repeats (CRISPR) and CRISPR-associated (Cas) systems provide adaptive immunity against mobile genetic elements (MGEs) in bacteria and archaea [1]. CRISPR arrays consist of an upstream promoter region (termed leader) followed by identical repeats that are separated by variable spacers originated from MGEs [2]. During the first stage of the CRISPR immunity, known as adaptation, short DNA fragments from MGEs are processed and incorporated into the host CRISPR arrays [3]. In the following stages, expression and interference, CRISPR arrays are transcribed and processed into mature CRISPR RNAs (crRNAs), which form ribonucleoprotein complexes with Cas proteins for crRNA-guided target recognition and degradation [4,5,6].

Cas1 and Cas2 are responsible for acquiring new spacers and are the most conserved Cas proteins [7]. Cas1 exhibits nuclease activity against singe-stranded, double-stranded, and branched DNAs, including Holliday junctions, replication forks and 5′-flaps [8,9,10]. The Cas1-Cas2 integrase complex directly catalyses nucleophilic attack of the protospacer at the leader end of the CRISPR array [11,12,13]. Crystal structures of Cas1-Cas2 complexes bound on both prespacer DNA with splayed ssDNA ends and the leader DNA have provided insights into prespacer selection, capture, and integration reaction in subtype I-E and type II-A systems [12,13,14,15]. One key question about CRISPR adaptation is how Cas1 and Cas2 are regulated to integrate new spacers specifically at the leader proximal sites. The integration host factor (IHF) is the only known factor required for specific spacer integration at the leader-proximal site in CRISPR-Cas subtype I-E [16,17,18] and I-F [19] systems. IHF binds to a consensus sequence and induces a DNA bending in the leader sequence, which offers a recognition motif for the recruitment of Cas1-Cas2 to the leader-proximal end. Moreover, the leader-proximal and the first repeat sequences also play important roles in specific spacer acquisition, especially for CRISPR-Cas systems lacking IHF homologs. For instance, a conserved leader-anchoring sequence identified in *Streptococcus pyogenes* subtype II-A CRISPR-Cas system specifies the site of spacer integration [20]. Similarly, in *Streptococcus thermophilus* subtype II-A CRISPR-Cas system, sequences within 10 bp of the integration site spanning both the leader and the first repeat are essential for spacer acquisition [21]. Furthermore, the leader-repeat junction and conserved motifs in the middle of the repeat were shown to be important for accurate spacer integration in subtypes I-B, I-E, and I-G CRISPR-Cas systems [22,23,24].

Although the leader proximal sequences were shown to be important for spacer acquisition, whether and how these sequences interact with the adaptation Cas are still open questions. In addition, it is unclear whether other *cis*- and *trans*-acting elements are involved in adaptation. Previously, we demonstrated that CRISPR adaptation is regulated by the CRISPR-associated factors Csa3a [25,26] and Csa3b [27] in the model organism *Sulfolobus islandicus* REY15A. In this study, we identified that a stem-loop structure is present at the leader-proximal end of the subtype I-A CRISPR-Cas system. We further demonstrated that Cas1 preferentially binds to the leader-proximal end with a stem-loop structure, and an accessory CRISPR factor modulated the integrase activity of Cas1 to avoid unexpected spacer acquisition outside the CRISPR array.

## 2. Results

### 2.1. Mutations at the Leader-Proximal End Reduces CRISPR Adaptation Efficiency

**Figure 1:**
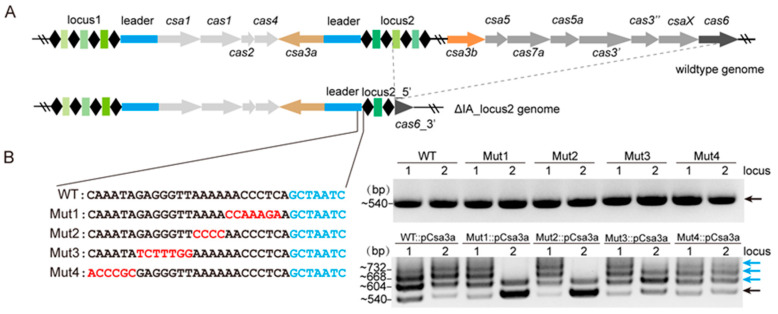
**Mutations at the leader-proximal end reduce spacer acquisition.** (**A**) Diagram of the type I-A CRISPR-Cas system in *S. islandicus* REY15A. The 206 bp leader sequences are identical in CRISPR locus1 and locus2 but located inverted. The type I-A *cas* genes and most of the CRISPR arrays were deleted in the ΔIA-locus2. Simultaneously, mutations identical to the sequences in Figure 1B were introduced into the leader of CRISPR locus2 to generate Mut1–4 mutant strains. Repeats are marked as black diamonds, and spacers are marked as green rectangles. (**B**) PCR amplification of the leader proximal regions of both CRISPR loci before (upper lanes) and after *csa3a* overexpression (carrying the *csa3a*-overexpression plasmid, pCsa3a) in WT or mutated strains (Mut1–4). For WT::pCsa3a and Mut4::pCsa3a, there are four main bands at both locus1 and locus2. For Mut1::pCsa3a and Mut2::pCsa3a, there are four main bands at locus1 but only two main bands at locus2. For Mut3::pCsa3a, there are four main bands at locus1 but three main bands at locus2. The bands corresponding to PCR products of the expanded arrays are indicated as blue arrows, and the black arrow indicates the parental bands. The sizes of the bands are indicated on the left. This result represents three independent spacer acquisition analyses.

The *S. islandicus* strain REY15A encodes two CRISPR loci with identical leaders but at an inverse transcriptional orientation (Figure 1A). Here, we deleted the whole interference module of subtype I-A CRISPR-Cas system, from the second spacer of locus2 to *cas6*, leaving only 138 bp of the 3′-end of the *cas6* gene (Figure 1A). Then, we introduced mutations at the leader regions of locus2 (the sequences as illustrated in Figure 1B) to compare the efficiencies of spacer acquisition between the wildtype and the mutated CRISPR loci. Adaptation was activated by inducing a plasmid-borne CRISPR-associated factor Csa3a, and spacer acquisition was monitored by PCR amplification of the leader-spacer regions [25]. We found that deletion of *cas6* and the subtype I-A interference module allowed the demonstration of super active *de novo* acquisition upon Csa3a induction (Figure 1B), as we reported previously [26]. In contrast, less expanded bands were obtained at locus2 compared with locus1 in the Mut1::pCsa3a and Mut2::pCsa3a strains (Figure 1B), indicating that the leader proximal region is crucial for spacer acquisition in vivo. There was a small decrease in the expanded bands for four main bands in locus2 and three main bands in locus1 in the Mut3::pCsa3a strain, indicating that this region modestly affects spacer acquisition (Figure 1B). Taken together, these results suggest that sequences of the leader-proximal end are important for CRISPR adaptation.

### 2.2. Cas1 Binds to the Leader-Proximal Sequences

**Figure 2:**
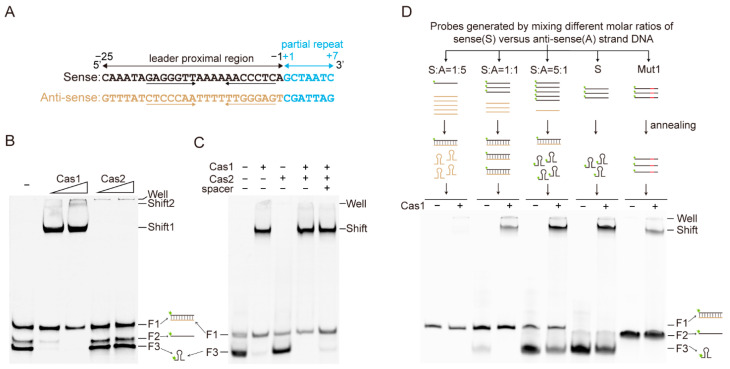
**Cas1 binds the stem-loop structure at the leader-proximal end**. (**A**) Sequence of the leader-proximal end in the subtype I-A CRISPR-Cas system of *S. islandicus* REY15A. The stem-loop structure is indicated by arrows. Numbers below indicate the locations of the leader sequence relative to the first repeat. (**B**) EMSA analysis of the binding between the Cas1 or Cas2 proteins and the 5′-end FAM fluorescent labeled leader-proximal probe. The probe sequences are shown in (**A**), and the reaction mixtures containing 0.3 μM probe and increasing concentration of Cas1 (0.6 μM and 1.2 μM) or Cas2 (3.3 μM and 6.7 μM) were loaded on the 12% native PAGE gel. (**C**) EMSA analysis of the effect of Cas2 and Cas2 with spacer on Cas1 binding to the 5′-end FAM fluorescent labeled leader-proximal probe. (**D**) EMSA analysis of Cas1 binding to different probes. These probes were generated through annealing of 5′-end FAM-labelled sense strand oligonucleotide (S) with unlabeled anti-sense strand oligonucleotide (**A**) at 1:5, 1:1 or 5:1 ratio, respectively. “S” indicates 5′-end FAM-labelled sense strand oligonucleotide, and “Mut1” indicates mutated 5′-end FAM-labelled sense strand oligonucleotide (the sequence is shown in Figure 1B).

Given that Cas1 and Cas2 specifically integrate spacers at the leader proximal site in different CRISPR-Cas systems [15,28], we hypothesized that Cas1 and/or Cas2 may recognize the leader-proximal ends for efficient spacer integration. Here, we focused on a proximal sequence at the 3′-end of the leader sequence in *S. islandicus* subtype I-A system (Figure 2A). We then tested whether this motif could be recognized by Cas1 or Cas2. A double-stranded (ds) DNA probe generated by annealing two complementary oligonucleotides (Figure 2A) was used for EMSA analysis. A strong retarded band was observed when the probe was incubated with Cas1, while no binding shift appeared even when Cas2 was added at higher concentrations (Figure 2B). Moreover, we also performed the EMSA experiment with Cas1-Cas2 and Cas1-Cas2-spacer complexes. However, there was no significant difference in the shift when the probe was incubated with Cas1-Cas2 complex, but there was a small increase in the free probe of dsDNA when incubated with the Cas1-Cas2-spacer complex, which may be due to the non-specific competition of the spacer with the probes (Figure 2C). This result indicates a direct binding of Cas1, but not Cas2, to the leader-proximal sequence of the *S. islandicus* subtype I-A CRISPR-Cas system.

Surprisingly, we observed that the free probe was separated into three bands on the native PAGE gel, to one of which Cas1 showed stronger binding efficiency (Figure 2B). These bands probably represented the forms of double-stranded, single-stranded, and single-stranded with stem-loop structure probes. Therefore, we designed different DNA probes corresponding to the three forms shown in Figure 2B, by annealing a 5′-end FAM-labelled sense strand oligonucleotide (S) with unlabeled antisense strand oligonucleotide (A) at different molar ratios. At a 1:5 molar ratio, all the labeled S oligo is present in dsDNA; while at a 1:1 molar ratio, the labeled S oligo is mostly in dsDNA form with a minority of ssDNA; and at a 5:1 molar ratio, the labeled S oligo is mostly present as ssDNA with a minority of dsDNA (Figure 2D). The EMSA results showed no retardation of the labeled probe in the 1:5 mixture group incubated with Cas1 (Figure 2D), suggesting no or very low binding between Cas1 and the dsDNA probe. This was further confirmed by EMSA using the 1:1 and 5:1 mixture groups, and only the sense strand probe representing the ssDNA probe was strongly shifted by Cas1, while the dsDNA probe was not retarded (Figure 2D). A strong retarded band appeared when using labeled S oligo as the probe, while a very weak band showed when the stem region was mutated in the Mut1 probe, and the free probes of S and Mut1 clearly migrated to different positions, indicating that stem-loop structure is important for Cas1 binding (Figure 2D). Taken together, these results indicate that Cas1 but not Cas2 specifically binds the leader-proximal sequence and prefers ssDNA with a stem-loop structure.

### 2.3. Cas1 Can Non-Specifically Bind to Stem-Loop Structured ssDNA

**Figure 3:**
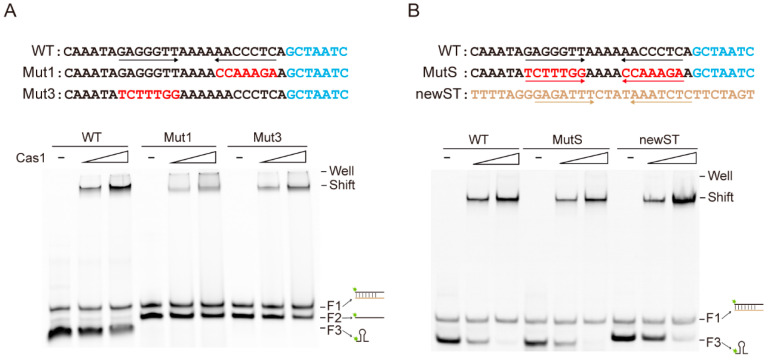
**Cas1 non-specifically binds to stem-loop structured ssDNA.** (**A**,**B**) EMSA analysis of Cas1 (0.6 μM and 1.2 μM) binding to the wildtype and the mutated probes (0.3 μM). These probes were generated through annealing of 5′-end FAM-labelled sense strand oligonucleotides and complementary unlabeled anti-sense strand oligonucleotides at 5:1 molar ratio, respectively. The sequences of Mut1 and Mut3 are shown in Figure 1B. MutS carries two mutated, and complementary, sequences at the stem region which occur individually in Mut1 and Mut3. The sequence of newST is completely different from the wildtype, but carries a similar stem-loop structure (7 bp stem with 4 nt loop).

It has been proposed that Cas1 integrase might be evolved from casposon (Figure S1A), a superfamily of DNA transposons using its endonuclease activity for integration into and excision out of the cellular genome, which is similar to the integration of spacers by the Cas1–Cas2 complex during spacer acquisition in CRISPR-Cas [29,30]. Further, the terminal inverted repeats (TIRs) of casposon share some similarity with the CRISPR repeat (Figure S1B), reminding us that stem-loop structure might be a general recognition motif for Cas1 in some subtypes of CRISPR-Cas systems. To further investigate how the stem-loop structure is important for Cas1 binding, we constructed two mutated probes (Mut1 and Mut3) for EMSA experiments (Figure 3A). The stem-loop structure is disrupted in Mut1 and Mut3 by introducing transversion mutations in each of the stem regions individually. The free probes of Mut1 and Mut3 migrated in the gel only in the forms of double strand and single strand, without the band corresponding to the ssDNA carrying the full stem-loop structure (Figure 3A). Accordingly, Mut1 and Mut3 showed weaker binding to Cas1 in comparison with the wild type probe (Figure 3A). To further investigate how Cas1 can bind the stem-loop structure ssDNA without sequence specificity, we constructed another two mutated probes (MutS and newST). Notably, MutS carries two mutated, and complementary, sequences at the stem region which occur individually in Mut1 and Mut3 (Figure 3B). In accordance with the restorage of the full stem-loop structure, MutS demonstrated the wild type binding with Cas1 (Figure 3B). Moreover, newST carries the same length and a similar 7 bp stem with 4 nt loop structure, but the sequences are quite different from the wild type probe. As expected, the intensity of Cas1 binding to the newST probe was as strong as that of the wild type probe. Overall, these results reveal that Cas1 non-specifically binds ssDNA with a stem-loop structure, and the specific sequences had a minimal effect on Cas1 binding ability.

We further studied whether Cas1 integrases from other systems can also bind the stem-loop structure on their leader-proximal ends. We demonstrated that Cas1 from *P. furiosus* bound the ssDNA probe of the leader-repeat which was predicted to carry a stable stem loop structure (Figure S1C), while mutation at the stem sequence strongly reduced the binding between Cas1 and the leader in the EMSA experiment (Figure S1D,E). Similarly, Cas1 integrase from *Synechrocystis* sp. bound the leader ssDNA (Figure S1G), which was predicted to carry two stem-loop structures (Figure S1C,F). We then conducted the EMSA experiment using a dsDNA probe by annealing the sense and antisense probe at a 5:1 molar ratio. We identified that Cas1 preferred the ssDNA with the stem-loop structure (Figure S1G). Moreover, we identified that Cas1 integrases from *P. furiosus* and *Synechrocystis* sp. bound other leaders with a stem loop structure (Figure S1H). Taken together, these results show that Cas1 integrases from other CRISPR-Cas systems might also recognize a stem-loop structure. However, it remains unclear whether the stem-loop structure is important for the integration activity of Cas1 in vivo.

### 2.4. Csa3a Interacts with Cas1 to Modulate Cas1 Integrase Activity

Given that Cas1 can bind any ssDNA with a stem-loop structure (Figure 2 and Figure S1), and there can be many stem-loop structures at a genome-wide scale, we propose that other factor(s) may modulate the integrase activity of Cas1 to avoid non-specific integration outside the CRISPR arrays. Therefore, an in vivo pull-down assay using plasmid-borne His-tagged Cas1 as the bait was conducted. The Cas1-binding proteins were eluted and identified by mass spectrometry analysis (Table S2). One of the identified proteins, Csa3a, is a transcriptional activator for adaptation *cas* genes [25] and the CRISPR arrays [26] through binding at the upstream site of the *csa1* promoter and a distal motif of the leader, respectively.

**Figure 4:**
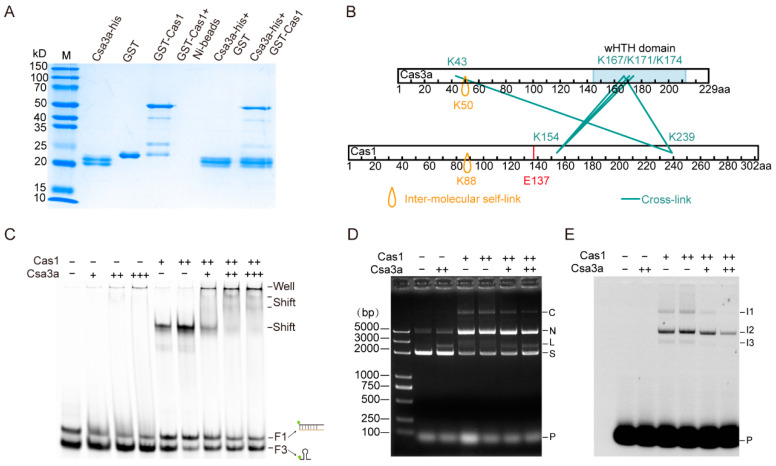
**Csa3a interacts with Cas1 to modulate Cas1 integrase activity.** (**A**) His-tag pull-down assay to analyze the interaction between Csa3a and Cas1. SDS-PAGE analysis of purified recombinant Csa3a-His, GST, GST-Cas1, and Ni-NTA column elution fractions of Csa3a-His incubated with GST or GST-Cas1. GST-Cas1 incubated with Ni-NTA beads was used as the control. Standard protein molecular mass (kDa) markers (M) are indicated on the left. (**B**) Cross-linking mass spectrometry to determine the interaction between Csa3a and Cas1. Inter-molecular self-links are indicated as orange drops. Cross-links between Csa3a and Cas1 are indicated as cyan lines. The red vertical line indicates the predicted nuclease active site E137 of Cas1 in *S. islandicus*; wHTH domain indicates the winged Helix-Turn-Helix domain. (**C**) EMSA analysis of Csa3a (0.6 μM, 1.2 μM, and 2.4 μM), Cas1 (0.6 μM and 1.2 μM), and 1.2 μM Cas1 with increasing amounts of Csa3a (0.6 μM, 1.2 μM, and 2.4 μM) binding to the wildtype probe. Well shift and super shifts appearing when Csa3a was added into the reaction. (**D**,**E**) in vitro integration of prespacers into the supercoiled plasmid using a 39 bp 5′-end FAM-labeled dsDNA as the prespacer. Addition of Cas1, Csa3a and the distal motif DNA (Csa3a-binding DNA) as a competitor are indicated above the gel images. Samples were separated by 1.5% agarose gel and visualized by EtBr staining (**D**) or fluorescent imaging (**E**). C: concatemer; N: nicked, L: linear; S: supercoiled; P: 5′-end FAM-labeled prespacer; I1, I2 and I3: integrated products.

First, in vitro pull-down was employed to confirm the interaction between Cas1 and Csa3a. We found that GST-tagged Cas1 and His-tagged Csa3a were co-eluted from the Ni-beads, while GST were not co-eluted with His-tagged Csa3a from the beads (Figure 4A), indicating that Csa3a specifically binds Cas1 in vitro. Then, we employed formaldehyde crosslinking of the Csa3a-Cas1 complex and LC-MS detection to identify interaction peptides between the two proteins. Five cross-linked peptides between Csa3a and Cas1 were detected (Figure 4B). We discovered that most of cross-link sites on Csa3a were located at the wHTH domain and the N-terminus (Figure 4B). Remarkably, the binding sites on Cas1 were found to be adjacent to the conserved integrase active site E137 residue [31] (Figure 4B).

The interaction between Csa3a and Cas1 prompted us to examine the effects of Csa3a on Cas1 binding ability and integrase activity. To investigate whether Csa3a influences Cas1 binding ability, we performed EMSA experiments with Cas1 and increasing amounts of Csa3a. As is shown in Figure 4C, two super shifts and a well shift appeared when Csa3a was added into the reaction, indicating that Csa3a interacts with Cas1 to form a larger Csa3a-Cas1-DNA complex. To study the impact of their interaction on Cas1 integrase activity, we performed in vitro integration assay with Cas1 and Csa3a according to the previously described method [31]. The preliminary results showed that there was a small decrease of the nicked form and an increase in the supercoiled form plasmid when Csa3a was added (Figure S2A). However, there was no obvious difference in PCR amplification of the integration sites between Cas1 only and Cas1 with Csa3a (Figure S2B). To clearly show the influence of Csa3a on integration, we used the CRISPR-containing plasmid and a 5′-end FAM-labelled dsDNA as the prespacer for in vitro integration. After separation of the in vitro integration products on the ethidium bromide (EtBr) strained agarose gel, we found that addition of Csa3a alone slightly transformed supercoiled plasmid DNA (band S) to the linear (band L) form (Figure 4D), and no integration products were identified by fluorescent imaging (Figure 4E). Cas1 alone strongly transformed the plasmid from supercoiled to nicked, as well as large, probably concatemeric, products (band C) (Figure 4D). Fluorescent imaging also showed three or more integration products in the presence of Cas1 (Figure 4E). However, addition of Csa3a into the integration reaction strongly reduced the signal of all integration products (Figure 4E), indicating that Csa3a inhibited non-specific integration of the Cas1 protein, most probably due to their interaction near the integrase active site (Figure 4B). Altogether, these results suggest that Csa3a modulates Cas1 integrase activity through interaction with Cas1.

### 2.5. Leader Motifs Disassociate Csa3a-Cas1 Interaction for Efficient CRISPR Adaptation

**Figure 5:**
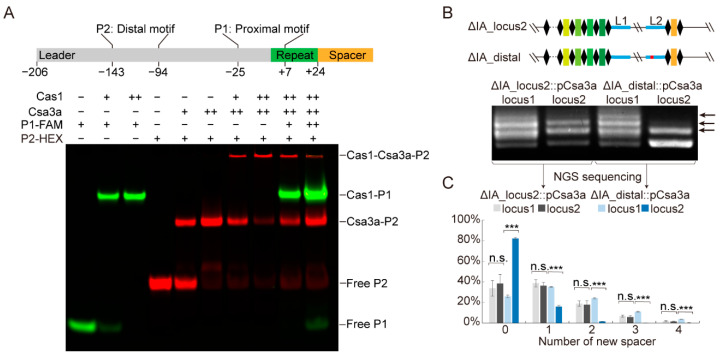
**Leader motifs disassociate Csa3a-Cas1 interaction for efficient CRISPR adaptation.** (**A**) EMSA assay to determine the competition effect of leader-proximal motif on the interaction between Csa3a and Cas1. Diagram of the Csa3a binding distal motif (−143~−94) and the Cas1 binding proximal motif (−25~+7) in the leader. Numbers are relative to the first nucleotide of the repeat sequence. The leader is shown in grey, and the CRISPR repeat is shown in green, followed by a spacer shown in orange. Lanes 1–3: 5′-end FAM-labeled proximal motif ssDNA with stem-loop structure (P1) was incubated with increasing amounts of Cas1. Lanes 4–6: 5′-end HEX-labeled distal motif dsDNA (P2) was incubated with increasing amounts of Csa3a. Lanes 7–8: P2 was incubated with Csa3a and increasing amounts of Cas1. Lanes 9–10: increasing amounts of P1 were added as the competitor for Cas1 binding. (**B**) PCR amplification for newly integrated spacers in both CRISPR loci after *csa3a* overexpression in ΔIA_locus2 strain (control) and ΔIA_distal (mutations introduced into the distal motif in the leader of locus2) strain. Diagram of the CRISPR loci of ΔIA_locus2 and ΔIA_distal is shown above. L1 and L2 represent the identical leaders of locus1 and locus2, respectively. Expanded bands relative to the new repeat-spacer units are indicated by arrows. (**C**) Proportion of new spacers obtained by analysing high-throughput sequencing of PCR products of both loci inΔIA_locus2 and ΔIA_distal strains. Error bars: standard derivations of three independent experiments. Statistical significance: n.s., non-significance; *** *p* < 0.001; two-way ANOVA and Dunnett.

Based on the above results that Cas1 binds to a proximal motif (Figure 2) and our previous result that Csa3a binds a distal motif on the leader [26], we wondered whether these two motifs affect the interaction between Csa3a and Cas1. To test this, we performed EMSA experiments using the 5′-end HEX-labeled leader-distal motif DNA as the probe (P2) and another 5′-end FAM-labelled leader-proximal motif DNA as the competition probe. As expected, the addition of Cas1 protein into the binding mixture of Csa3a formed a larger Cas1-Csa3a-DNA complex, as reflected by the further retarded band on the EMSA gel (Figure 5A and Figure S3B). This is consistent with the previous result that addition of Csa3a into the Cas1 binding reaction formed the Csa3a-Cas1-DNA complex (Figure 4C). Moreover, when the 5′-end FAM-labelled leader-proximal motif DNA (P1) was added to compete for Cas1 binding in the reaction, the retarded band disassociated into one Csa3a-P2 shift and another Cas1-P1 shift, indicating that the Csa3a-Cas1 complex was forced to separate in the presence of the two leader motifs (Figure 5A). This result suggests that not only the proximal motif but also the distal motif is crucial for spacer acquisition. To further assess the in vivo function of the leader-distal motif, we introduced transversion mutations into the distal motif that were previously found to abolish the binding with Csa3a in vitro [26]. These mutations strongly reduced the adaptation efficiency at the adjacent CRISPR locus2, but not at locus1 carrying the intact distal motif, as detected by PCR analysis (Figure 5B). High-throughput sequencing of the PCR products showed that the distal motif mutation led to a 2-fold reduction in the uptake of a single new spacer, and a 15- to 200-fold reduction in the uptake of two to four new spacers at locus2 when compared with adaptation at locus1 (Figure 5C). Together, these results demonstrate that efficient CRISPR adaptation at the CRISPR array requires recognition of the leader-distal motif by the CRISPR-associated factor Csa3a.

## 3. Discussion

Although previous studies have demonstrated that DNA motifs in the leader-proximal end or leader-repeat junction determined site-specific spacer integration [20,21,24,32], how exactly these motifs function was an open question. Similarly, how the adaptation Cas is recruited to the leader region remained largely unknown. The only known example was the recruitment of adaptation Cas by IHF in subtype I-E and I-F systems. IHF was found to bind at the leader-proximal region and deform the leader to provide a favourable conformation for the recognition of the Cas1-Cas2 complex [18,33]. It appears that the IHF mediated recruitment of adaptation Cas is an exception because only a few microorganisms encode the *ihf* gene. However, IHF recognizes other DNA elements which share high identity to the IHF binding motif, increasing the efficiency of non-canonical spacer integration near these DNA motifs in vivo [34]. Most importantly, the *ihf* gene is encoded in only few species, suggesting that there might be other unknown mechanisms modulating spacer integration into the CRISPR array in diverse CRISPR-Cas systems.

In this study, we have demonstrated that the leader-proximal region is crucial for spacer acquisition in the subtype I-A model system of *S. islancicus* Rey15A (Figure 2). Furthermore, we have identified a stem-loop structure located in the leader-proximal region which is preferred for Cas1 binding in vitro (Figure 3). Stem-loop structures are targets for binding by many proteins, such as helicase, integrase, and endonuclease, which are involved in many significant DNA metabolism processes, including replication, transcription, recombination, and DNA repair [35]. In *E. coli*, Cas1 is reported to recognize a cruciform DNA, and a palindromic motif present in the repeat is important for the specific interaction of Cas1-Cas2 with a CRISPR locus [9,36]. Additionally, an in vitro integration assay has also found the most frequent integration site in the control plasmid, which is located at an inverted repeat sequence adjacent to a AT-rich promoter [37]. All these previous results suggest that unique DNA structures play a critical role in spacer acquisition, and our results further confirm that Cas1 preferentially binds to a stem-loop structure at the leader-proximal end in the subtype I-A CRISPR-Cas system. However, Cas1 protein could bind any ssDNA with a similar stem-loop structure without sequence specificity in vitro (Figure 3 and Figure S1), leading us to identify an accessory factor Csa3a in *S. islandicus*. Csa3a interacts with Cas1 and binds a distal motif on the leader, modulating the integrase activity of Cas1 (Figure 4). This is further supported by the fact that mutation at the distal Csa3a binding site significantly reduced CRISPR adaptation efficiency (Figure 5B,C).

**Figure 6:**
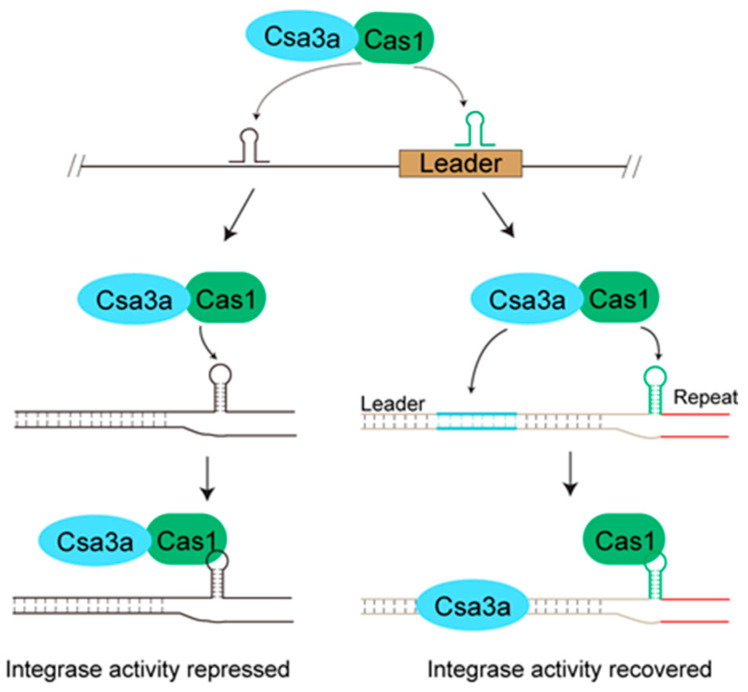
**A proposal for Cas1 binding DNA structure and modulation of integrase activity by a CRISPR regulator.** Csa3a can form a complex with Cas1 to inhibit the integrase activity but not the DNA binding activity of Cas1. The Csa3a-Cas1 complex can bind to some sites with a specific stem-loop structure in the genome. When Csa3a-Cas1 complex binds to the sites outside the CRISPR array, the integrase activity is repressed to avoid atypical spacer integration. Only when the complex encounters the leader does the integrase activity recover by Cas1 binding to the stem-loop structure at the proximal end and Csa3a binding to the distal motif.

Based on our results in this study and the previously reported data [25,26], we propose a model for Cas1 binding and modulation of Cas1 integrase activity by an accessory factor. In this model, there may be some stem-loop structures existing in the genome in some cases which can be non-specifically bound by Cas1 (Figure 6). To avoid atypical spacer integration outside of the CRISPR array, an accessory factor, Csa3a for example, is employed to interact with Cas1 to repress its integration activity. Meanwhile, Csa3a is a transcriptional factor for activation of CRISPR transcription by binding to a distal motif on the leader [26]. When the Csa3a-Cas1 complex translocated to the neighbourhood of the leader, Cas1 preferentially binds to the proximal motif with a stem-loop structure and Csa3a simultaneously binds to the distal motif, liberating Cas1 to recover the integrase activity (Figure 6). This model also suggests other factor(s), besides Csa3a, in different CRISPR-Cas systems could function similarly to regulate the integrase activity of Cas1, or recruit the adaptation complex to the leader-proximal end.

## 4. Materials and Methods

### 4.1. Strains, Growth Conditions and Transformation of Sulfolobus

*S. islandicus* E233*S* (Δ*pyrEF*Δ*lacS*) was used as the parental strain for all the genetic manipulations. All *Sulfolobus* strains, including wildtype (E233*S*) and the derivatives were cultured at 78 °C in the SCV medium, or SCVU medium (SCV medium + 20 μg/mL uracil), or ACV inducible medium [38]. Plasmids for genome editing or *csa3a*-overexpression were transformed into *S. islandicus* E233*S* cells by electroporation, and transformants were selected on two-layer phytal gel plates, as described previously [38].

### 4.2. Protein Expression and Purification

The *cas1*, *cas2* and *csa3a* genes from *S. islandicus* REY15A, *cas1* genes from *Pyrococcus furiosus* COM1 and *Synechocystis* sp. PCC6803 were amplified from their genomic DNA using the primers listed in Table S1 and cloned into the pET30a or pGEX-6P-1 expression plasmids. *E. coli* DH5α and Rosetta cells were used for gene cloning and recombinant protein production, respectively. *E. coli* Rosetta cells for protein expression were grown at 37 °C in LB medium until OD_600_ = 0.6–0.8, and then induced overnight with the addition of 0.5 mM IPTG at 16 °C. The expression and purification of the Cas1 and Cas2 proteins with C-terminal His tag were conducted as described previously [25]. For purification of GST-tagged Cas1, Rossetta cells were harvested, resuspended, and lysed in binding buffer (10 mM Tris-HCl, pH 8.0, 1 mM EDTA, 150 mM NaCl). After centrifugation at 14,000 rpm at 4 °C for 30 min, the soluble fraction was filtered (pore size 0.22 μm Millex-GP Syringe Filter Unit, Coolwind, Guangzhou, China) and incubated with Glutathione-Sepharose 4FF beads (GE Healthcare, United States). GST-tagged protein was eluted in the elution buffer (50 mM Tris-HCl, pH 9.0, 20 mM glutathione). Dialyzed proteins were concentrated, flash frozen and stored at −80 °C before use.

### 4.3. Electrophoretic Mobility Shift (EMSA) Assays

The 5′-FAM-labeled single-stranded probes were synthesized by Tsingke Biotechnology Co., Ltd. (Beijing, China), and double-stranded DNA probes were generated by annealing the oligonucleotides with one of 5′-end FAM-labelled (Table S1). Detailed EMSA assays were carried out as described previously [25].

### 4.4. Prediction of Stem-Loop Structure and IHF Binding Sites

The 40 bp DNA sequences of the 3′-end of leaders were extracted for stem-loop prediction using the “UNAFold Web Server” (http://www.unafold.org/DNA_form.php (accessed on 21 January 2022)) with its default settings [39].

### 4.5. Construction of S. islandicus Mutant Strains

The endogenous CRISPR-based genome editing method [40] was employed to construct *S. islandicus* mutant strains. A type I-A protospacer adjacent motif (CCN) and the immediately downstream 40 nt DNA sequence (protospacer) were selected as target sites on the *cas6* gene. A pair of complementary oligonucleotides matching the selected protospacer were then designed and synthesized (Table S1). The spacer fragment was generated by annealing of the two oligonucleotides and inserted into a *Sulfolobus* CRISPR-cloning vector (pSe-Rp) [41] at the BspMI sites, yielding the interference plasmid pAC-*cas6*. Then, upstream and downstream DNA fragments were amplified from *S. islandicus* REY15A genomic DNA using two pairs of primers (mutL-F-Sal I/mutL-R and mutR-F/mutR-R-Not I). The two PCR fragments were fused by overlapping PCR using the primer pair of mutL-F-Sal I/mutR-R-Not I, yielding donor DNA for homologous recombination. After gel purification, 10 ng of this donor DNA was used as the template in a second round of two-step overlapping PCR as described above (primers listed in Table S1) to generate donor DNA with mutations of interest. The resulting donor DNA fragments were digested with Sal I and Not I, and inserted into the pAC-*cas6* plasmid separately, resulting in the editing plasmids: pGE-ΔIA_locus2, pGE-ΔIA_Mut1, pGE-ΔIA_Mut2, pGE-ΔIA_Mut3, pGE-ΔIA_Mut4, and pGE-ΔIA_distal to yield the mutation strains ΔIA_locus2 (deletion of type I-A *cas* genes and most of the CRISPR arrays of locus2), ΔIA_Mut1–4 strains (deletion of type I-A *cas* genes and most of the CRISPR arrays of locus2 and mutations introduced into the leader of CRISPR locus2) and ΔIA_distal (deletion of type I-A *cas* genes and most of the CRISPR arrays of locus2 and mutations introduced into the leader-distal motif of locus2), respectively. These pGE plasmids were then transformed into *S. islandicus* E233*S* individually by electroporation. Correct transformants were selected and confirmed by PCR amplification of pGE plasmids and DNA sequencing of the mutated regions. Subsequently, transformants were plated on the SCV plates containing 5-FOA and uracil to remove the pGE plasmids. Then, the *csa3a*-overexpression plasmid pCsa3a [25] was introduced into the cells by electroporation to activate spacer acquisition. Three colonies of each mutation strain carrying pCsa3a plasmid were used in subsequent experiments.

### 4.6. His-Tag Pull-Down Assay

Equal amounts of purified GST-Cas1 or GST and Csa3a-His proteins were mixed with Ni-NTA agarose beads (Thermo Fisher) and incubated on a rotating platform for 1 h at 4 °C. Then agarose beads were collected by centrifugation at 3000× *g* for 3 min at 4 °C and washed three times with the wash buffer (20 mM HEPES-NaOH [pH 7.6], 500 mM NaCl, 20 mM imidazole). The remaining protein complexes were eluted with the elution buffer (20 mM HEPES-NaOH [pH 7.6], 500 mM NaCl, 500 mM imidazole). Samples of the eluted proteins were then subjected to SDS–PAGE, followed by Coomassie Brilliant Blue R-250 staining.

For the in vivo pull-down assay, the *cas1* gene was amplified and cloned into *S. islandicus* vector pSeSD [38], resulting in a pCas1 expression plasmid. Then the plasmid was transformed into *S. islandicus* cells for protein expression. The cells were grown at 78 °C in the SCV medium until OD_600_ = 1.0. The cells were harvested, resuspended, and lysed in binding buffer (20 mM HEPES-NaOH [pH 7.6], 500 mM NaCl). After centrifugation at 14,000 rpm at 4 °C for 30 min, the soluble fraction was filtered and incubated with Ni-NTA agarose beads. Then beads were washed with the wash buffer (20 mM HEPES-NaOH [pH 7.6], 500 mM NaCl, 100 mM imidazole) until no protein was detected. The remaining protein complexes were eluted with the elution buffer (20 mM HEPES-NaOH [pH 7.6], 500 mM NaCl, 500 mM imidazole) and subjected to mass spectrometry analysis.

### 4.7. In Vivo Spacer Acquisition Assay and High-Throughput Sequencing

The spacer acquisition assay was performed as previously described [25]. High-throughput sequencing of the PCR products of the leader-proximal regions and sequencing data analysis were conducted as described previously [42]. All high-throughput sequencing data have been deposited at the Sequence Read Archive (SRA) database under the BioProject accession number PRJNA782792.

### 4.8. In Vitro Integration Assay

The prespacer was generated by the annealing of two oligonucleotides or with one FAM-labelled at the 5′-end if required. The DNA fragment of the mini-CRISPR (leader-repeat-spacer1-repeat) was PCR amplified and cloned into the T-vector (Takara, Dalian, China) to generate the pCRISPR plasmid for the in vitro integration assay. in vitro integration assays employing Cas1 and Csa3a were performed as previously described [31] with some modifications. Different concentrations of Cas1 or Csa3a (as described in the figure legends) were incubated individually or together at 55 °C for 30 min. Then the solution was added into the reaction containing 500 nM prespacer DNA, 100 ng plasmid DNA, 1 μL 10× integration buffer (200 mM Tris [pH 7.5], 100 mM NaCl), 1 μL MnCl_2_ (50 mM), and appropriate water making the total reaction volume up to 10 μL. This reaction was incubated at 55 °C for 30 min and then quenched with 1 μL of proteinase K (20 mg/mL; Tiangen Biotech, Beijing, China) at 37 °C for 1 h. After phenol extraction, the products were mixed with 10 × DNA loading dye and separated on 1% agarose gel in 1× TAE buffer. The gel was scanned for fluorescence using a FUJIFILM scanner (FLA-5100). Finally, the gel was stained with ethidium bromide and visualized by the Bio-Rad GelDocXR+ imaging system. For the PCR amplification, 1 μL of the extracted solution was added to a PCR reaction containing 1 μL of forward and reverse primer (Dup-F and M13-F), 10 μL 2X Taq Master Mix and 7 μL water. The PCR reaction was performed at 95 °C for 5 min, followed by 32 cycles of 95 °C for 30 s, 50 °C for 30 s and 72 °C for 30 s, with a final extension at 72 °C for 2 min. The PCR products were separated on 1.2% agarose gel in 1× TAE buffer, and the gel was stained and visualized as previously described.

### 4.9. Cross-Linking Mass Spectrometry

Cross-linking mass spectrometry was performed as previously described with some modifications [43]. Two samples of 10 μg of purified Csa3a and Cas1, dissolved in 20 mM HEPES-NaOH (pH 7.6), 500 mM NaCl, and 5% glycerol were incubated together for 20 min at 40 °C. Then 1 mM (final concentration) of the cross-linker solution (BS3, Thermo Scientific Pierce) was added to the protein sample at a 1:1 (*w*/*w*) ratio, mixed well and cross-linked at room temperature for 1 h. The cross-linking reaction was quenched with 50 mM NH_4_HCO_3_ solution at room temperature for 15 min. The proteins were precipitated by adding five volumes of pre-cooled acetone, and stored at −20 °C for 2 h followed by centrifugation at 14,000× *g* at 4 °C for 10 min. Then sediment was washed by adding five volumes of pre-cooled methanol and centrifuging at 14,000× *g* at 4 °C for 10 min. The sediment was resuspended in 10 μL 8 M urea solution (dissolved in 50 mM NH_4_HCO_3_), followed by adding 15 μL 100 mM DTT and incubating at 37 °C for 30 min, and then 15 μL 100 mM IAM was added and the solution was placed in the dark for 30 min. The final protein sample was diluted in lysis buffer (50 mM NH_4_HCO_3_, 1 mM CaCl_2_) to bring the concentration of urea to 2 M, and then digested in solution using trypsin (Promega) overnight at 37 °C. The reaction was quenched with formic acid solution to a 1% final concentration and desalted by Stage Tip. The obtained fractions were dried by vacuum centrifugation to near complete dryness and resuspended in 0.1% FA water. Then an appropriate amount was injected into the EASY-nLC™ 1200 system (Thermo Fisher Scientific, Rockford, USA) to identify cross-linked peptides. The data analysis was performed with the XLinkX node incorporated into Proteome Discoverer (v.2.3). The visualization of the detected cross-links was performed on xiNET (http://crosslinkviewer.org/upload.php (accessed on 20 July 2021)).

## Data Availability

The data presented in this study are openly available in the Sequence Read Archive (SRA) database under the BioProject accession number PRJNA782792. (https://dataview.ncbi.nlm.nih.gov/object/PRJNA782792).

## Supplementary Materials

The following supporting information can be downloaded at: https://www.mdpi.com/article/10.3390/ijms231710178/s1.
