# Supplementary material for: DNA Motifs and an Accessory CRISPR Factor Determine Cas1 Binding and Integration Activity in Sulfolobus islandicus
Source: Int J Mol Sci. 2022 Sep 5;23(17):10178. doi: 10.3390/ijms231710178 (PMC9456107; doi:10.3390/ijms231710178)
Supplement: Supplementary file 1 [file ijms-23-10178-s001.zip › ijms-1898755-supplementary.pdf]

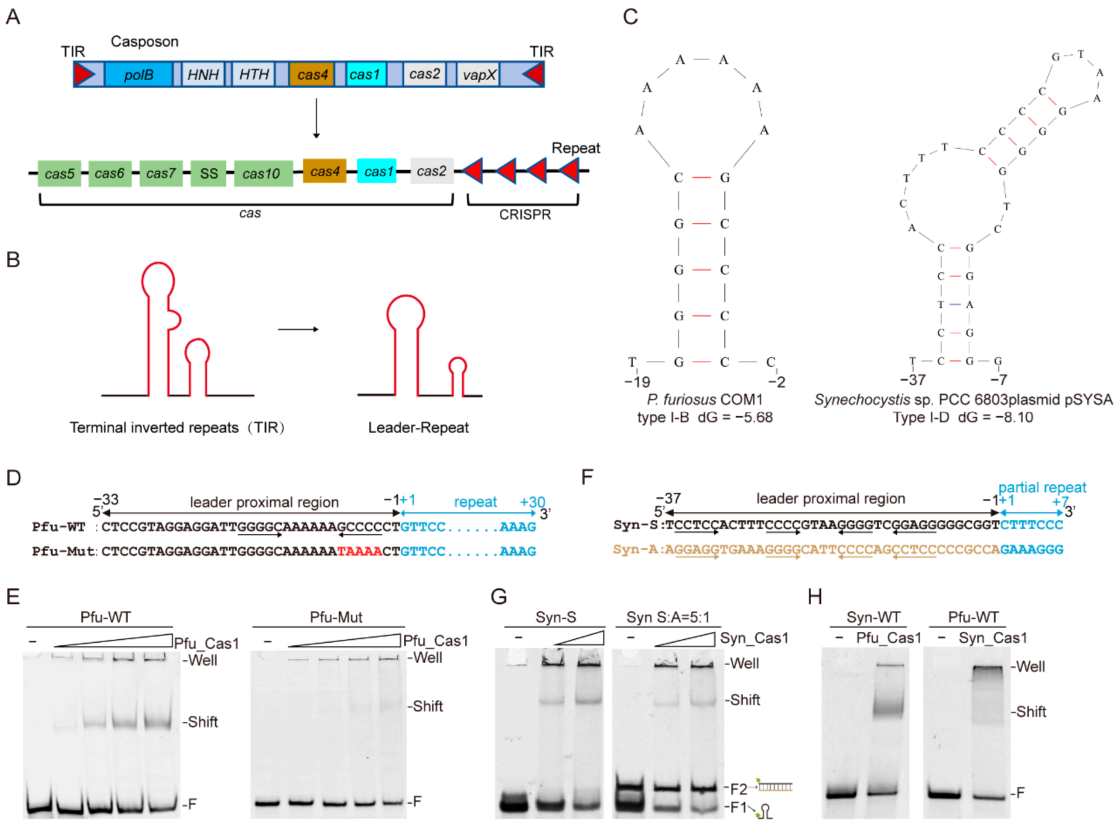

Figure S1. A hypothesis of the evolution of CRISPR-Cas from casposon and identification of Cas1 from *Pyrococcus furiosus* COM1 and *Synechocystis* sp. PCC 6803 binding their leaders with a possible stem-loop structure. (**A**) The evolution of the CRISPR-Cas system from a casposon. The *cas1* gene is shown in light blue. The Cascade genes are shown in green. (**B**) Terminal invertied repeats (TIRs) are palindromic, which share some similarity with the CRISPR leader-repeat. (**C**) Predicted stem-loop structures at the leader-proximal sites of the *Pyrococcus furiosus* COM1 and *Synechocystis* sp. PCC 6803. The stem-loop structures were predicted using the "UNAFold Web Server" and the free energy (dG) of each sequence was calculated automatically. (**D**) leader-repeat sequences from *P. furiosus* COM1. (**E**) EMSA experiments showing PfuCas1 bound the ssDNA probe of *P. furiosus* leader-repeat (Pfu-WT) and the leader-repeat mutant (Pfu-Mut), respectively. (**F**) leader-repeat sequences from *Synechocystis* sp. PCC 6803. (**G**) EMSA experiments showing SynCas1 bound the ssDNA probe of *Synechocystis* sp. PCC 6803 leader-repeat (Syn-S) and the leader-repeat mutant (Syn-Mut), and the dsDNA probe (Syn S:A=5:1), respectively. (**H**) EMSA experiments showing PfuCas1 bound the ssDNA probe of *Synechocystis* sp. PCC 6803 leader-repeat (Syn-S), and SynCas1 bound the ssDNA probe of *P. furiosus* leader-repeat (Pfu-WT), respectively. The palindromic sequences were indicated by arrows and mutations which destroyed the stem-loop were indicated by red letters. F: free probe.

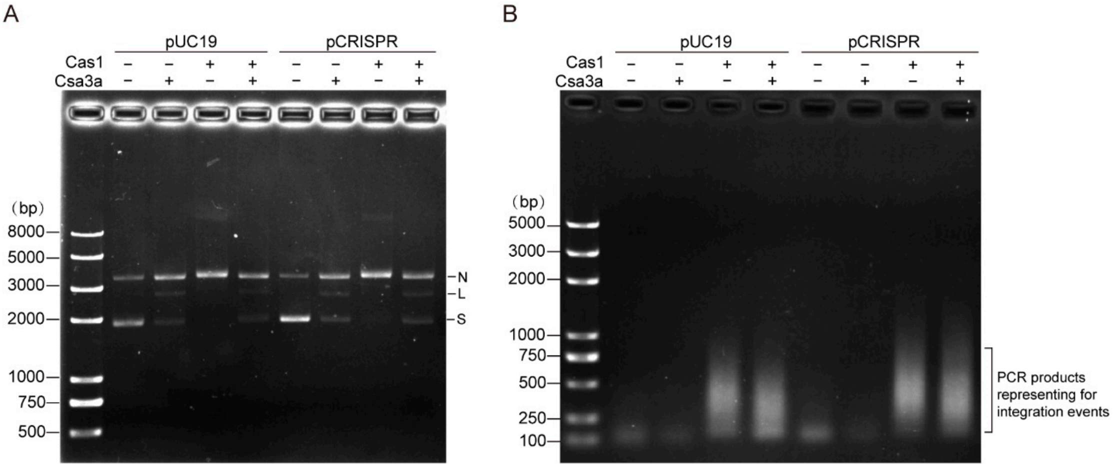

Figure S2. *In vitro* integration of spacers into the supercoiled plasmids. (**A**) Agarose gel analysis to identify the *in vitro* integration of a duplex DNA substrate into the supercoiled pUC19 and pCRISPR (pUC19 with an insertion sequence of leader-repeat) plasmids. Cas1 and/or Csa3a were incubated with 100 ng plasmids and 2 μM 39 bp dsDNA at 55°C for 30 mins. The products were separated on a 1.5% agarose gel and stained with ethidium bromide. N: nicked, L: linear; S: supercoiled. (**B**) PCR amplification of the purified products from (A) to detect integration. A primer against the plasmid and a primer of the duplex substrate were used to amplify the DNA integrated into the plasmid. The smeared PCR products corresponding to integration events taking place at different sites.

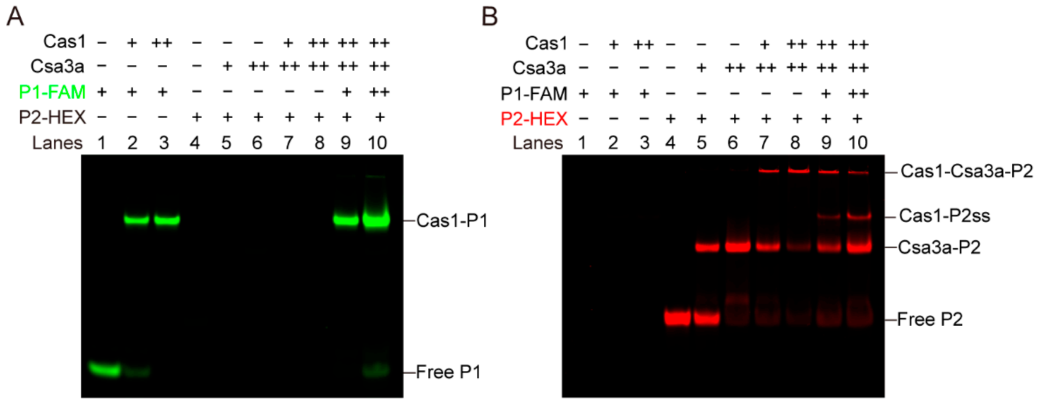

Figure S3. EMSA assay to determine the competition of leader-proximal motif on the interaction between Csa3a and Cas1. A 32 nt of 5'-end FAM-labeled proximal motif ssDNA with stem-loop structure was used as Probe 1 (P1), and a 50 bp of 5'-end HEX-labeled distal motif dsDNA was used as Probe 2 (P2). The same gel was scanned for fluorescence of FAM (A) and HEX (B) separately. Lane 1-3: 0.5 μM P1 was incubated with increasing amounts of Cas1 (0, 0.6, 1.2 μM), Lane 4-6: 0.5 μM P2 was incubated with increasing amounts of Csa3a (0, 1.0, 2.0 μM), Lane 7-8: 0.5 μM P2 was incubated with 2.0 μM Csa3a and increasing amounts of Cas1(0.6, 1.2 μM), Lane 9-10: increasing amounts of P1 (0.5, 1.0 μM) was added as the competitor for Cas1 binding. The band of Cas1-P2ss might represent that Cas1 bound the trace HEX-labeled ssDNA of the distal motif with a stem-loop structure.

Supplementary Table S1: primers used in this study

**Primers and nucleotides**	**Sequence (5′ to 3′)**
**Primers for cloning**	
Siscas1-F-BamH I	CGGGATCCATTAAGACCTTAATAATATCGG
Siscas1-R-Sal I	ACGCGTCGACCTACAAAACTAGTTTAAATCCGT
Siscas1-F-NdeI	CGCCATATGAAGACCTTAATAATATCGG
Siscas1-R-NotI	ATTGCGGCCGCCAAAACTAGTTTAAATCCGT
Siscas2-F-NdeI	CGCCATATGAAAATAATTGTAGTTTATG
Siscas2-R-NotI	ATTGCGGCCGCCACTAACCATTCTCCCACAACC
Siscsa3a-F-NdeI	CGCCATATGAAGTCATACTTTGTAACTTTGG
Siscsa3a-R-NotI	ATTGCGGCCGCTTGAATTTCATTTTCTATATATAATTTTG
Pfucas1-F-NdeI	CGCCATATGAGAAAAAAGTCTTTAACAAT
Pfucas1-R-NotI	TATGCGGCCGCAAACCACGCGACAAGGGG
Syncas1-F-NdeI	CGCCATATGAGAACGCTCTATGTGT
Syncas1-R-NotI	TATGCGGCCGCGCGGATTTCGTAGGGAG
cas6-spacer-F	AAAGCTACATTGCTTTCGTCTAAGGTTTTACTTCCTCCTTCCCT
cas6-spacer-R	TAGCAGGGAAGGAGGAAGTAAAACCTTAGACGAAAGCAATG TAG
mutL-F-Sal I	CGCGTCGACTCCGAACTGGTGAAAGATAC
mutL-R	GTTAACAAATACTTCAAAACATTTTTGCGAATTAGTTTGA
mutR-F	TCAAACTAATTCGCAAAAATGTTTTGAAGTATTTGTTAAC
mutR-R-Not I	ATTGCGGCCGCTATTATTACTTTGATCTT
mutL-Mut1-R	GATTAGCTTCTTTGGTTTTAACCCTCTATTTGATCTTTTCTTCTC CC
mutR-Mut1-F	AAAACCAAAGAAGCTAATCTACTATAGAATTGAAAGACAC
mutL-Mut2-R	GGGGAACCCTCTATTTGATCTTTTCTTCTCCC
mutR-Mut2-F	GATCAAATAGAGGGTTCCCCAACCCTCAGCTAATCTACTA
mutL-Mut3-R	TTTTCCAAAGATATTTGATCTTTTCTTCTCCC
mutR-Mut3-F	GATCAAATATCTTTGGAAAAAACCCTCAGCTAATCTACTA
mutL-Mut4-R	TTTTAACCCTCGCGGGTATCTTTTCTTCTCCCTTTCT
mutR-Mut4-F	GATACCCGCGAGGGTTAAAAAACCCTCAGCTAATCTACTA
mutL-distal-R	GAGGAAATGGGGGTCCCGTTCGCTTAAGGAATTCTTCATAAA GATAATG
mutR-distal-F	CGAACGGGACCCCCATTTCCTCGTAAAAAAGATAAGAAAGAG TAATATAATA
mini-CRISPR-F	CGCGTCGACTTGTTTTCCTTTTTCCTTGTTTCG
mini-CRISPR-R	CGGGATCCATTTTTGCGAATTAGTTTGA
**Primers for amplification of the leader proximal CRISPR regions**	
leader-F1	CTTGTTTCGTTTTGGGTTAGGTTGT
locus1S4-R	TATATTTGGTCCATAGGAGGACCAG
locus2-cas6R-R	AAGTCTGAGGAAGTATATTTACCCT

leader-F2	GGGAGAAGAAAAGATCAAATAGAGG
locus1S2-R	AAAGATTGAAATTCTTAAAGCTTACAAGAG
locus2S2-R	GTTAACAAATACTTCAAAACATTTTTGCGAATTAGTTTGA
**Primers for EMSA and *in vitro* integration**	
leader-WT-F	FAM-CAAATAGAGGGTTAAAAAACCCTCAGCTAATC
leader-WT-R	GATTAGCTGAGGGTTTTTTAACCCTCTATTTG
leader-Mut1-F	FAM-CAAATAGAGGGTTAAAACCAAAGAAGCTAATC
leader-Mut1-R	GATTAGCTTCTTTGGTTTTAACCCTCTATTTG
leader-Mut3-F	FAM-CAAATATCTTTGGAAAAAACCCTCAGCTAATC
leader-Mut3-R	GATTAGCTGAGGGTTTTTTCCAAAGATATTTG
leader-MutS-F	FAM- CAAATATCTTTGGAAAACCAAAGAAGCTAATC
leader-MutS-R	GATTAGCTTCTTTGGTTTTCCAAAGATATTTG
newST-F	FAM-TTTTAGGGAGATTTCTATAAATCTCTTCTAGT
newST-R	ACTAGAAGAGATTTATAGAAATCTCCCTAAAA
leader-P1-F	FAM-AGATCCCGGGAAAACCCGGGAATAGTAA
leader-P1-R	TTACTATTCCCGGGTTTTCCCGGGATCT
Pfu-leader-F	FAM-<br>CTCCGTAGGAGGATTGGGGCAAAAAAGCCCCCTGTTCCAATAA<br>GACTACAAAAGAATTGAAAG
Pfu-leader-Mut-F	CTCCGTAGGAGGATTGGGGCAAAAAATAAAACTGTTCCAATAA<br>GACTACAAAAGAATTGAAAG
Syn-leader-WT-F	FAM-<br>TCCTCCACTTTCCCCGTAAGGGGTCGGAGGGGGCGGTCTTTCCC
Syn-leader-WT-R	GGGAAAGACCGCCCCCTCCGACCCCTTACGGGGAAAGTGGAGG<br>A
Syn-leader-Mut-F	FAM-<br>TCCTCCACTTTCCCCGTAATTTTTCTTCTTGGGCGGTCTTTCCC
Dup-F-FAM	FAM- CGAGCTCGCCATGGTGAGCACAGAGGATAATGTAACACT
Dup-F	CGAGCTCGCCATGGTGAGCACAGAGGATAATGTAACACT
Dup-R	AGTGTTACATTATCCTCTGTGCTCACCATGGCGAGCTCG
M13-F	TGTAAAACGACGGCCAGT

Restriction sites are underlined.

Supplementary Table S2. Mass spectrometry analysis identified CRISPR-associated proteins co-purified with Cas1 in *S. islandicus* REY15A.

Gene ID	Description	Coverage	Peptides	PSMs	Unique Peptides	Found in Sample
SiRe_0760	Csa1	51.94346	13	21	13	High
SiRe_0761	Cas1	54.13793	17	29	16	High
SiRe_0763	Cas4	25.71429	4	5	4	High
SiRe_0764	Csa3a	22.76786	5	6	5	High
SiRe_0766	Csa5	16.08392	2	2	2	High
SiRe_0767	Cas7	20.24922	5	6	5	High
SiRe_0768	Cas5	5.416667	1	1	1	High
SiRe_0769	Cas3	5.788423	3	3	3	High
SiRe_0771	Casx	8.695652	2	2	2	High
SiRe_0598	Cmr2	2.89296	3	3	3	High
SiRe_0600	Cmr1	1.890756	1	1	1	High
SiRe_0603	Cmr7	41.95402	5	7	5	High

Coverage: percentage of the protein sequence covered by identified peptides. Peptides: number of peptides found in the sample. PSMs: score of peptide spectrum matches. Unique Peptides: number of unique peptides found in the sample.
